# Retraction: Design, synthesis, and quorum quenching potential of novel catechol–zingerone conjugate to find an elixir to tackle *Pseudomonas aeruginosa* through the trojan horse strategy

**DOI:** 10.3389/fchem.2025.1754947

**Published:** 2025-12-04

**Authors:** Frontiers Editorial Office

The journal retracts the 15 June 2022 article cited above.

Following publication, concerns were raised regarding similarities between Figures 3C, 5C, 6, 8B, 9A in this paper and figures published in other publications to describe different reagents or experiments.

The authors failed to provide a satisfactory explanation during the investigation, which was conducted in accordance with Frontiers’ policies. As a result, the data and conclusions of the article have been deemed unreliable, and the article has been retracted.

This retraction was approved by Chief Editors of *Frontiers in Chemistry* and the Chief Executive Editor of Frontiers. The authors did not agree with this retraction.

Frontiers would like to thank Dr. Yuki Ichinose for contacting the journal regarding the published article.

